# Locus of control moderates the association of COVID-19 stress and general mental distress: results of a Norwegian and a German-speaking cross-sectional survey

**DOI:** 10.1186/s12888-021-03418-5

**Published:** 2021-09-06

**Authors:** Henning Krampe, Lars Johan Danbolt, Annie Haver, Gry Stålsett, Tatjana Schnell

**Affiliations:** 1 Department of Anesthesiology and Operative Intensive Care Medicine (CCM, CVK), Charité - Universitätsmedizin, corporate member of Freie Universität Berlin, Humboldt-Universität zu Berlin, and Berlin Institute of Health, Berlin, Germany; 2 Practical Theology, MF Norwegian School of Theology, Religion and Society, Oslo, Norway; 3 Centre for Psychology of Religion, Innlandet Hospital Trust, Ottestad, Norway; 4 Faculty of Social Sciences, Norwegian School of Hotel Management, University of Stavanger, Stavanger, Norway; 5 Faculty of Social Sciences, School of Psychology, University of Wollongong, Wollongong, NSW Australia; 6 Social Sciences, MF Norwegian School of Theology, Religion and Society, Oslo, Norway; 7 Modum Bad Psychiatric Center, Vikersund, Norway; 8 Existential Psychology Lab, Institute of Psychology, University of Innsbruck, Innsbruck, Austria

**Keywords:** Anxiety, COVID-19, Depression, Locus of control (LoC), Moderator analysis, Pandemic, PHQ-4

## Abstract

**Background:**

An internal locus of control (LoC I) refers to the belief that the outcome of events in one’s life is contingent upon one’s actions, whereas an external locus of control (LoC E) describes the belief that chance and powerful others control one’s life. This study investigated whether LoC I and LoC E moderated the relationship between COVID-19 stress and general mental distress in the general population during the early months of the COVID-19 pandemic.

**Methods:**

This cross-sectional survey study analysed data from a Norwegian (*n* = 1225) and a German-speaking sample (*n* = 1527). We measured LoC with the Locus of Control-4 Scale (IE-4), COVID-19 stress with a scale developed for this purpose, and mental distress with the Patient Health Questionnaire 4 (PHQ-4). Moderation analyses were conducted using the PROCESS macro for SPSS.

**Results:**

The association between COVID-19 stress and general mental distress was strong (r = .61 and r = .55 for the Norwegian and the German-speaking sample, respectively). In both samples, LoC showed substantial moderation effects. LoC I served as a buffer (*p* < .001), and LoC E exacerbated (p < .001) the relation between COVID-19 stress and general mental distress.

**Conclusions:**

The data suggest that the COVID-19 pandemic is easier to bear for people who, despite pandemic-related strains, feel that they generally have influence over their own lives.

An external locus of control, conversely, is associated with symptoms of depression and anxiety. The prevention of mental distress may be supported by enabling a sense of control through citizen participation in policy decisions and transparent explanation in their implementation.

**Supplementary Information:**

The online version contains supplementary material available at 10.1186/s12888-021-03418-5.

## Background

Numerous studies found that mental distress has substantially increased during the COVID-19 pandemic, and first systematic reviews and meta-analyses reported prevalence rates ranging from 21.8 to 33.0%, from 22.0 to 33.7%, and from 34.4 to 41.1%, for clinically relevant anxiety, depression, and general mental distress, respectively [1–8]. Researchers observed that people experienced not only mental distress but also stress that was directly related to the pandemic and its aftermath [9, 10]. Thus, several scales were developed to measure stress specifically due to the pandemic. These COVID-19 stress scales primarily assess symptoms of anxiety and fears associated with COVID-19, but also various other facets of stress experience during the pandemic, such as feeling restricted by lockdown measures, uncertainty and doubts of how to protect oneself and loved ones against infections, sleep disturbance, confusion, frustration, anger, loneliness, social isolation, and fears of the future [11, 12]. While there is ample evidence that higher COVID-19 stress is significantly related to symptoms of mental distress [12–17], we do not know much about factors influencing this relationship. In particular, there is a need for investigations into resilience factors and resources that can buffer the effect of acute COVID-19 stress on mental distress. Research on resilience and resources will help inform public health measures and interventions to improve coping with stressful experience during the current pandemic and its aftermath, and it will provide important insights for dealing with future crises.

Until now, several factors were found to serve as resources buffering the effects of stressors, stressful experiences or risk factors on mental health or protective health promoting behaviour during the first year of the pandemic. Among these moderating factors are higher self-esteem [18], greater psychological flexibility and acceptance of difficult experience [19], higher meaning in life and self-control [12], less digital emotion contagion [20], higher age [21], male gender and lower COVID-19 stress [22], emotion regulation by cognitive reappraisal strategies [23], increased resilience [24], trust in the healthcare system [25], identifying positive over negative aspects of COVID-19 lockdown [26], as well as utilizing prenatal care services [27]. Although their stress buffering and/or resilience strengthening effects are empirically confirmed in the respective study samples, it is open to what extent the protective mechanisms of the mentioned moderators would work in other samples and across various circumstances. Research is needed to identify psychological moderators that are stable, established, and robust, so that their assumed stress-buffering effect would be less dependent on sample characteristics and regional pandemic differences.

Locus of control (LoC) is among the four most widely investigated personality traits [28]. It is a relatively stable dimension that describes the extent to which individuals are convinced to be able to control their environment and future, and to experience significant events as consequences of their own behaviour [29]. LoC covers two aspects. While external LoC refers to the belief that chance and powerful others control one’s life, internal LoC describes the belief that the outcome of events in one’s life is contingent upon one’s actions. LoC has originally been assessed with continuous internal-external scales (e. g. [29, 30]), whereas later on, separate scales for internal and external LoC were considered more appropriate (e.g. [31–33]). Cross-sectional and longitudinal studies from various international regions found that higher internal LoC and lower external LoC were moderately associated with greater mental health, lower situational stress, and lower mental distress, like depression and anxiety [30, 34–38]. Studies on psychosocial factors during the COVID-19 pandemic also investigated the role of LoC. In a sample of 339 participants from the United States, Berg & Lin (2020) examined predictors of the self-rated likelihood to engage in COVID-19 prevention behaviors. While internal health-related LoC did not show significant associations, external health-related LoC regarding powerful others predicted preventative behaviors [39]. Two studies examined associations of LoC and mental health. In a sample of 1723 adults from the USA and five European countries, Sigurvinsdottir et al. (2020) found significant negative correlations of internal LoC, and significant positive correlations of external LoC with depression, anxiety, and stress. Multiple regression analyses with diverse predictors showed that higher external LoC was moderately related to higher depression, anxiety and stress, while higher internal LoC was slightly related to lower depression and less stress, but not to anxiety [40]. In a sample of 667 participants from India, Alat et al. (2021) investigated the protective role of psychological resources for mental health. Higher internal LoC correlated moderately with higher positive affect and affect balance, as well as with lower negative affect and psychological distress. Using confirmatory factor analysis and path analysis, the authors found a small indirect effect indicating that affect balance mediated the association between internal LoC on psychological distress [41]. With its established validity for more than five decades, a temporal and transnational robustness, as well as replicated associations with perceived stress and mental health, LoC seems to be a promising candidate when it comes to factors that might attenuate the putative relationship between COVID-19 stress and general mental distress.

The objective of this study was therefore to investigate whether internal LoC and external LoC moderated the relation between COVID-19 stress and general mental distress during the early months of the COVID-19 pandemic. In order to assess the robustness of the assumed stress-buffering (LoC I) and stress-exacerbating (LoC E) effects, we collected data in a Norwegian and a German-speaking sample (primarily from Germany and Austria). Norway on the one hand and the central-European countries Germany and Austria on the other hand showed both differences and similarities in pandemic-relevant aspects of country and societal characteristics, as well as regarding the extent of the pandemic. Similarities existed with regard to the timing, extend, and strictness of national COVID-19 restriction guidelines [42, 43]. Differences can be seen in the following characteristics: Most importantly, population density is substantially lower in Norway, and institutional trust, as in the other Scandinavian countries, is higher than in Austria and Germany [42, 44]. According to data from the Johns Hopkins University (2021), in the first three weeks of March 2020 Norway had more cumulative confirmed COVID-19 cases per million people than Germany and Austria, but from April 1, 2020 to the end of our survey period, the number of cumulative confirmed cases per million people was always lower in Norway than in Germany and Austria [45]. The same holds for the cumulative number of confirmed COVID-19 deaths. Despite of the contextual variations, we expected that in both samples, high internal LoC would buffer, and high external LoC would exacerbate the association between COVID-19 stress and general mental distress.

## Methods

This cross-sectional survey was conducted in a Norwegian sample during the weeks when the strict COVID-19 regulations were gradually eased (May, 7, 2020 to June, 4, 2020 [44]), and in a German-speaking sample during the times of strict regulations and in the weeks thereafter (Austria, Germany, April 10, 2020 to May 28, 2020 [12]). Participation was voluntary, without compensation, and could be terminated by participants anytime. Ethical approval was issued by the Review Board (Psychology) of the University of Innsbruck, No 09/2020, as well as by Personvernombudet Innlandet Hospital Trust, Norway, No 20/02104–1. All participants expressed their informed consent by explicitly agreeing to continue with the questionnaire after being informed about the study’s aims, employed data protection, participants’ rights, and contact points for questions or concerns.

Data were collected by means of convenience sampling, using online questionnaire tools. Invitations to the study were sent out via university, business, worldview-related and regional network newsletters, and posted in several newspapers and news websites.

### Participants

The inclusion criteria of this study were a minimum age of 18 years, agreement to participant consent, and completion of the questionnaire. Cases with disproportionately short response times were deleted (*n* = 2 and *n* = 7 for the Norwegian and the German-speaking sample). After exclusion, the total sample amounted to *N* = 2752. The Norwegian sample (*n* = 1225) included mainly participants with Norwegian nationality (95.5%) and some with Swedish (1.4%), Danish (0.7%), and other nationalities (2.4%). The German-speaking sample (*n* = 1527) included participants with German (51.9%), Austrian (37.5%), Italian (5.8%), and other nationalities (4.8%). Demographic and psychological characteristics of the participants are shown in Table 1.

**Table 1:** Demographic and psychological characteristics of study participants; mean [SD]; n (%)

	Norwegian sample *n* = 1209-1225	German-speaking sample *n* = 1522-1527	***p***
Age (years)	50.26 [13.16]	40.35 [16.66]	<.001
Gender			<.001
Women	897 (73.20)	993 (65.00)	
Men	326 (26.60)	528 (34.60)	
Divers	2 (0.20)	6 (0.40)	
Relationship status			<.001
Married/partnered	683 (55.80)	953 (62.40)	
Other	542 (44.20)	574 (37.60)	
Children			<.001
No	252 (20.60)	975 (63.90)	
Yes	971 (79.40)	552 (36.10)	
Living together status			.460
Living alone	249 (20.30)	328 (21.50)	
Living with others	976 (79.70)	1199 (78.50)	
Education:			<.001
Secondary	19 (1.60)	190 (12.40)	
Advanced	133 (10.90)	453 (29.70)	
University	1073 (87.60)	884 (57.90)	
I have been infected with Sars CoV-2			
Yes	14 (1.10)	12 (0.80)	
No / I do not know	1211 (98.90)	1515 (99.20)	
A close person has been infected			.336
Yes	136 (11.10)	130 (8.50)	
No / I do not know	1089 (88.90)	1397 (91.50)	.022
LoC ^a)^			
Internal	3.65 [0.87]	3.94 [0.81]	<.001
External	1.84 [0.75]	2.31 [0.86]	<.001
COVID-19 stress ^b)^	1.34 [0.82]	1.54 [0.89]	<.001
General mental distress ^c)^	2.51 [2.35]	3.48 [2.82]	<.001
Elevated general mental distress ^c)^			
> 5	111 (9.10)	291 (19.10)	<.001
> 3	355 (29.00)	628 (41.10)	<.001
> 2	551 (45.00)	861 (56.40)	<.001
Depression ^c)^	1.38 [1.33]	1.82 [1.52]	<.001
Elevated depression (> 2) ^c)^	175 (14.30)	346 (22.70)	<.001
Anxiety ^c)^	1.12 [1.28]	1.66 [1.57]	<.001
Elevated anxiety (> 2) ^c)^	123 (10.00)	321 (21.00)	<.001

^a^LoC measured by the IE-4 subscales LoC Internal and LoC External (range: 1–5)

^b^Acute psychological stress due to COVID-19 measured by the COVID-19 stress scale (range: 0–5)

^c^General mental distress, depression, and anxiety measured by the PHQ-4. Total scale (range 0–12) with cut-offs > 5, > 3, > 2 for at least severe, moderate, and mild distress. Subscales depression (PHQ-2) and anxiety (GAD-2) (range 0–6), with cut-offs > 2 for elevated depression and anxiety

#### Measures

##### Locus of control (LoC)

The 4-item Internal/External Locus of Control-4 Scale, IE-4, [32] was used to assess LoC. The subscales for internal LoC (LoC I) and external LoC (LoC E) consist of two items each, describing beliefs of personal control with a range from 1 (does not apply at all) to 5 (applies completely). Kovaleva et al. (2012) report extensive data on good psychometric properties of the German version, including content, factorial, and construct validity [32]: Reliabilities for two normative samples were determined by McDonald’s omegas of .71 and .70 for LoC I, and of .63 and .53 for LoC E. A confirmatory factor analysis included the IE-4 and the KMKB, a German LoC scale that is based on a short version of the Levenson locus of control scale. The results showed that corresponding latent factors of both scales correlated highly between .92 and .99. Concerning construct validity, the authors found positive correlations of the LoC I scale with self-efficacy (.61), life satisfaction (.53), optimism (.36), and persistence (.37), as well as negative correlations of the LoC E scale with self-efficacy (.32), life satisfaction (−.48), optimism (−.32), and persistence (−.22). In the present study, McDonald’s omegas of LoC I and LoC E were .69 and .71 (Norwegian sample), and .80 and .60 (German-speaking sample). For use in the Norwegian sample, the original items were translated and a back-translation checked.

##### COVID-19 stress

Because there were no instruments available at that time, we developed a novel scale to determine the extent of acute psychological stress due to COVID-19 [12]. After examining the relevant literature and drawing on population surveys released by the media, we generated seven items tapping a broad range of affective stress reactions (feelings of intolerability, boredom, anger, and being left alone) and fears and pessimism about internal resources and the future. The items are displayed in suppl. Table 1 [Supplementary Material]. With a view to the current situation, items are rated on a six-point Likert scale ranging from 0 (strongly disagree) to 5 (strongly agree). For use in the Norwegian sample, the original items were translated and a back-translation checked. Internal consistencies in the present study were good, with McDonald’s omega coefficients of .81 (Norwegian sample) and .81 (German-speaking sample). Confirmatory factor analyses (CFAs) support a one-dimensional model of COVID-19 stress in both samples (Χ^2^ (14) = 101.83, RMSEA = .072, SRMR = .043, CFI = .943 for the Norwegian sample, and Χ^2^ (14) = 133.63, RMSEA = .075, SRMR = .044, CFI = .946 for the German-speaking sample). Evidence of the construct validity of the scale can be inferred from the first study of the German-speaking sample [12]. The COVID stress scale showed a correlation of .5 with general mental distress, as measured with the sum score of the PHQ-4. This correlation is substantial enough to indicate the assumed shared variance, and small enough to suggest that two different constructs are being measured here. Furthermore, the COVID-19 stress scale showed correlations with psychological measures that corresponded to published relationships between those and other stress measures [46]: Meaningfulness (−.28), crisis of meaning (.41), and self-control (−.21). As reported for the established Coronavirus Anxiety Scale (CAS) [14], also our COVID-19 scale showed a small negative correlation with age (.21, *p* < .001).

##### General mental distress

We measured mental distress with the Patient Health Questionnaire 4, PHQ-4, [47, 48], a brief four-item measure of core symptoms of depression and anxiety. It uses a four-point Likert scale ranging from 0 (not at all) to 3 (nearly every day). Participants were asked to respond to the items with a view to the past two weeks. Studies from the last decade reported Cronbach alpha values between .78 and .85 and established construct validity and factorial validity of the PHQ-4 [47, 49–51]. Several findings have confirmed its validity as a measure of general mental distress, with correlations of around .70 with established indicators of general mental distress (e.g. [47, 50–52]). A recent study found that the PHQ-4 sum score and the sum score of the PHQ-ADS, a 16-item combination of the PHQ-9 and GAD-7, correlated comparably strong with other indicators of general mental distress [51]. The PHQ-4 has also demonstrated good reliability and validity in clinical and population samples for the Norwegian and German versions (e.g. [47–50, 53, 54]). McDonald’s omegas in this study were .91 (Norwegian sample), and .91 (German-speaking sample). Several cut-off points have been validated with > 2, > 3, > 5 indicating mild, moderate, and severe mental distress [47, 50].

##### Demographics and living conditions

The sociodemographic section assessed participants’ age, gender, relationship status, children, living together status, education, and personal experiences with Sars-CoV-2 infections. The specific categories of the demographic variables are shown in Table 1.

#### Statistical analyses

Descriptive results are expressed as relative frequencies in percent, as well as means and standard deviations. Comparisons of the two samples were performed using chi-square tests for categorical data and t-tests for continuous data. The reliability coefficient McDonald’s omega was calculated with IBM SPSS AMOS 25 Graphics, as described by Hayes & Coutts [55]. For all statistical tests, a two-tailed *p*-value ≤.05 was considered statistically significant. Due to their small number, data from participants identifying as gender divers were excluded from analyses that contained gender as a variable.

Moderation analyses were conducted using the PROCESS macro, version 3.5 [56, 57] for SPSS, version 25 [58]. Multiple linear regression models tested whether the independent variables COVID-19 stress, internal LoC, external LoC, and the interaction between COVID-19 stress and internal and external LoC, respectively, had statistically significant associations with general mental distress as measured by the total score of the PHQ-4. In a further step, these moderation analyses were repeated including the covariates age, gender, relationship status, children, living together status, and education.

The statistical interaction between the independent variables ‘COVID-19 stress’ on the one hand and ‘internal LoC’ and ‘external LoC’ on the other hand indicated whether individual differences in LoC moderated individual differences of severity of mental distress in participants with varying severity of acute COVID-19 stress. In order to probe the interactions, analyses using the Johnson-Neyman technique were conducted for all eight regression models (four regression analyses by two samples) [57]. The Johnson-Neyman technique calculates the statistical significance of the effect of an independent variable, in this study COVID-19 stress, for all values of the moderator variable, in this study internal or external LoC. Thus, the Johnson-Neyman technique can ‘identify points of transition along the continuum of the moderator between a statistically significant and nonsignificant effect of X’ [57, page 13]. The resulting ranges of the values of the moderator where the independent variable is significantly associated with the dependent variable are called regions of significance.

## Results

### Sample characteristics and zero-order correlations

The two study samples differed significantly concerning demographic and psychological characteristics (Table 1). Compared with the German-speaking sample (*n* = 1527), the Norwegian sample (*n* = 1225) was older and had higher percentages of women, of people with children, and of people with university education. The Norwegian sample had lower scores of both internal and external LoC, lower COVID-19 stress, as well as lower general mental distress, depression, and anxiety. Correspondingly, compared with the German-speaking participants, the Norwegian sample had lower rates of clinically significant depression (14.3% versus 22.7%), anxiety (10.0% versus 21.0%), and severe general mental distress (9.1% versus 19.1%). While there were small but statistically significant differences in relationship status and Sars CoV-2 infection rates of close persons, the samples did not differ regarding living together status and personal Sars CoV-2 infection rates.

Table 2 displays the intercorrelations between COVID-19 stress, general mental distress, LoC I, and LoC E. In both samples, COVID-19 stress and general mental distress had large positive correlations. All other correlations were of moderate to small size, with LoC I and LoC E correlating negatively with each other, and COVID-19 stress and general mental distress correlating negatively with LoC I, and positively with LoC E. The psychological variables were only weakly associated with demographic characteristics, however the majority of these correlations reached statistical significance in both samples (Table 3), suggesting to include the demographic variables into adjusted moderation analyses of the psychological variables. No significant correlations were found between psychological variables and experiences with Sars CoV-2 infections.

**Table 2:** Correlations between locus of control (LoC), COVID-19 stress, and general mental distress

	LoC I ^a)^	LoC E ^a)^	COVID-19 stress ^b)^
**a.** Norwegian sample (*n* = 1225)			
LoC E	−.22^***^		
COVID-19 stress	−.11^***^	.32^***^	
General mental distress ^c)^	−.14^***^	.32^***^	.61^***^
**b.** German-speaking sample (n = 1527)			
LoC E	−.41^***^		
COVID-19 stress	−.15^***^	.25^***^	
General mental distress ^c)^	−.31^***^	.35^***^	.55^***^

*** p < .001

^a)^LoC measured by the IE-4 subscales LoC Internal and LoC External (range: 1–5)

^b)^Acute psychological stress due to COVID-19 measured by the COVID-19 stress scale (range: 0–5)

^c)^General mental distress measured by the PHQ-4 (range 0–12)

**Table 3:** Correlations between demographic variables and LoC, COVID-19 stress, and general mental distress

	LoC I ^h)^	LoC E ^h)^	COVID-19 stress ^i)^	General mental distress ^k)^
**a.** Norwegian sample (n = 1209 -1225)				
Age (years)	−.06	−.10^**^	−.12^***^	−.19^***^
Gender ^a)^	.03	−.06^*^	.03	.05
Relationship status ^b)^	−.13^***^	−.01	−.11^***^	−.12^***^
Children ^c)^	−.03	−.06	−.15^***^	−.19^***^
Living together status ^d)^	−.05	.04	−.10^***^	−.10^***^
Education ^e)^	−.02	−.07^*^	−.08^**^	−.11^***^
Infected Sars CoV-2 ^f)^	−.04	−.02	−.02	−.01
Close person infected with Sars CoV-2 ^g)^	−.02	−.002	.02	−.02
**b.** German-speaking sample (*n* = 1521-1527)				
	LoC I ^h)^	LoC E ^h)^	COVID-19 stress ^i)^	General mental distress ^k)^
Age (years)	−.20^***^	.09^**^	−.21^***^	−.17^***^
Gender ^a)^	.06^*^	.00	.11^***^	.06^*^
Relationship status ^b)^	−.09^**^	−.07^**^	−.13^***^	−.12^***^
Children ^c)^	−.10^***^	.09^***^	−.14^***^	−.14^***^
Living together status ^d)^	.15^***^	−.01	−.05^*^	−.06^*^
Education ^e)^	−.02	−.11^***^	−.08^**^	−.10^***^
Infected Sars CoV-2 ^f)^	.03	−.03	−.01	−.02
Close person infected with Sars CoV-2 ^g)^	.04	−.01	−.004	−.03

*p < .05, **p < .01, *** p < .001

^a)^ 1 = male, 2 = female

^b)^ 0 = not partnered; 1 = married/partnered

^c)^ 0 = no children, 1 = children

^d)^ 0 = living alone, 1 = living with somebody

^e)^ 0 = secondary/advanced, 1 = university

^f)^ 0 = not infected with Sars CoV-2/ do not know, 1 = infected with Sars CoV-2

^g)^ 0 = close person not infected with Sars CoV-2/ do not know, 1 = close person infected with Sars CoV-2

^h)^ LoC measured by the IE-4 subscales LoC Internal and LoC External (range: 1–5)

^i)^ Acute psychological stress due to COVID-19 measured by the COVID-19 stress scale (range: 0–5)

^k)^ General mental distress measured by the PHQ-4 (range 0–12)

### Moderation analyses

**Fig. 1:**
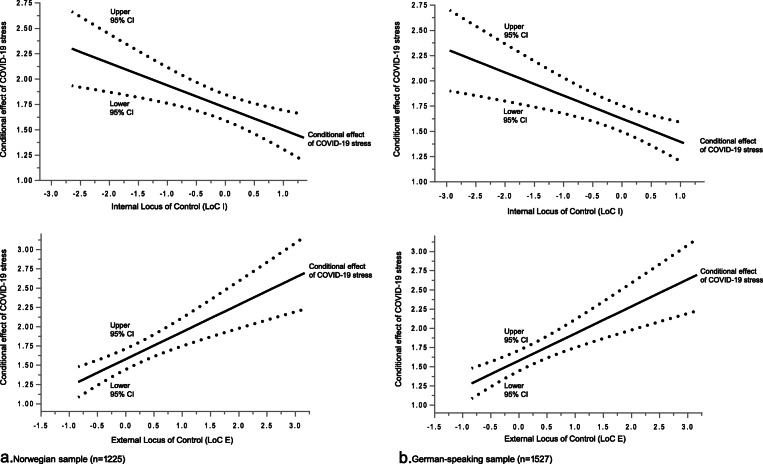
**a, b.** Johnson-Neyman plots of the interaction between COVID-19 stress and LoC. Moderators: LoC I (upper graphs), LoC E (lower graphs). The black continuous lines show the conditional effects of COVID-19 stress on general mental distress (PHQ-4) for all values of LoC, and the dotted lines above and below indicate the corresponding 95% confidence intervals (CI)

Table 4 shows the results of multiple regression models analysing the prediction of general mental distress (PHQ-4 sum score). COVID-19 stress, LoC I, LoC E, and the interaction between COVID-19 stress and LoC I and LoC E, respectively, had statistically significant independent effects on general mental distress in both the Norwegian and German-speaking samples. Higher COVID-19 stress, lower LoC I, and higher LoC E predicted higher general mental distress. The significant interactions indicated moderation effects. While higher LoC I buffered the effect of COVID-19 stress on general mental distress, higher LoC E exacerbated the effect. Figures 1a and b display plots of Johnson-Newman analyses to illustrate the interactions of COVID-19 stress and LoC I and LoC E, respectively. With increasing scores of LoC I, the conditional effect of COVID-19 stress on general mental distress decreased. With rising scores of LoC E, it increased. The conditional effects were significant for the total range of scores of LoC I and LoC E. Finally, Table 4 shows that all associations between the psychological variables and general mental distress remained significant when the moderation analyses were adjusted for the demographic variables age, gender, relationship status, having children, living together status, and education.

**Table 4:** Simple moderation: LoC moderates COVID-19 stress predicting general mental distress

**a. Unadjusted moderation**	Norwegian sample (n = 1225)			German-speaking sample (n = 1527)		
**Regression analysis 1: Internal locus of control (LoC I)**						
	*Coeff (SE) [95% CI]*	*t*	*p*	*Coeff (SE) [95% CI]*	*t*	*p*
Intercept	2.49 (0.05) [2.38; 2.59]	46.93	<.001	3.46 (0.06) [3.35; 3.57]	59.45	<.001
COVID-19 stress (IV)	1.72 (0.07) [1.59; 1.85]	26.31	<.001	1.63 (0.07) [1.50; 1.76]	24.66	<.001
LoC I (Mod)	−0.21 (0.06) [−0.33; − 0.09]	−3.40	<.001	− 0.75 (0.07) [− 0.89; − 0.61]	−10.32	<.001
COVID-19 stress x LoC I	−0.22 (0.07) [− 0.35; − 0.09]	−3.23	.001	−0.23 (0.07) [− 0.36; − 0.10]	−3.39	<.001
**Regression analysis 2: External locus of control (LoC E)**						
	*Coeff (SE) [95% CI]*	*t*	*p*	*Coeff (SE) [95% CI]*	*t*	*p*
Intercept	2.44 (0.05) [2.33; 2.54]	45.13	<.001	3.43 (0.06) [3.32; 3.55]	57.87	<.001
COVID-19 stress (IV)	1.58 (0.07) [1.45; 1.71]	23.18	<.001	1.55 (0.07) [1.42; 1.68]	22.99	<.001
LoC E (Mod)	0.39 (0.07) [0.25; 0.54]	5.28	<.001	0.71 (0.07) [0.57; 0.84]	10.05	<.001
COVID-19 stress x LoC E	0.35 (0.08) [0.20; 0.50]	4.65	<.001	0.27 (0.07) [0.13; 0.41]	3.83	<.001
**b. Adjusted moderation**	Norwegian sample (*n* = 1205)			German-speaking sample (*n* = 1516)		
**Regression analysis 1: Internal locus of control (LoC I)**						
	*Coeff (SE) [95% CI]*	*t*	*p*	*Coeff (SE) [95% CI]*	*t*	*p*
Intercept	3.84 (0.36) [3.14; 4.55]	10.69	<.001	4.24 (0.34) [3.57; 4.90]	12.51	<.001
COVID-19 stress (IV)	1.63 (0.07) [1.50; 1.76]	24.66	<.001	1.52 (0.07) [1.39; 1.66]	22.32	<.001
LoC I (Mod)	−0.26 (0.06) [−0.38; −0.14]	−4.30	<.001	−0.84 (0.08) [−0.99; −0.70]	−11.21	<.001
COVID-19 stress x LoC I	−0.20 (0.07) [− 0.33; − 0.06]	−2.91	.004	−0.23 (0.07) [− 0.36; − 0.10]	−3.44	<.001
Age (years)	−0.02 (0.01) [− 0.03; − 0.01]	− 4.04	<.001	−0.01 (0.01) [− 0.02; − 0.00]	− 2.55	.011
Gender ^a)^	0.21 (0.12) [− 0.03; 0.44]	1.75	.080	0.00 (0.12) [− 0.24; 0.25]	0.03	.979
Relationship status ^b)^	0.01 (0.13) [−0.25; 0.26]	0.04	.969	0.06 (0.14) [−0.22; 0.33]	0.40	.689
Children ^c)^	−0.22 (0.16) [− 0.53; 0.09]	−1.39	.166	− 0.31 (0.16) [− 0.62; 0.01]	−1.88	.060
Living together status ^d)^	− 0.26 (0.16) [− 0.58; 0.06]	− 1.62	.105	−0.00 (0.17) [− 0.33; 0.33]	−0.02	.983
Education ^e)^	−0.45 (0.16) [− 0.77; − 0.14]	− 2.82	.005	−0.33 (0.12) [− 0.57; − 0.10]	− 2.81	.005
**Regression analysis 2: External locus of control (LoC E)**						
	*Coeff (SE) [95% CI]*	*t*	*p*	*Coeff (SE) [95% CI]*	*t*	*p*
Intercept	3.65 (0.36) [2.95; 4.34]	10.28	<.001	4.23 (0.34) [3.56; 4.90]	12.44	<.001
COVID-19 stress (IV)	1.50 (0.07) [1.37; 1.64]	21.81	<.001	1.46 (0.07) [1.32; 1.59]	20.82	<.001
LoC E (Mod)	0.39 (0.07) [0.25; 0.54]	5.31	<.001	0.75 (0.07) [0.61; 0.89]	10.51	<.001
COVID-19 stress x LoC E	0.37 (0.08) [0.22; 0.51]	4.88	<.001	0.28 (0.07) [0.15; 0.42]	3.99	<.001
Age (years)	−0.02 (0.01) [−0.03; −0.01]	−3.73	<.001	−0.01 (0.01) [− 0.02; 0.001]	−1.83	.067
Gender ^a)^	0.24 (0.12) [0.01; 0.47]	2.01	.045	−0.02 (0.13) [− 0.27; 0.22]	−0.18	.855
Relationship status ^b)^	0.02 (0.13) [−0.23; 0.27]	0.16	.870	0.05 (0.14) [−0.22; 0.33]	0.38	.706
Children ^c)^	−0.22 (0.16) [− 0.53; 0.08]	−1.44	.151	− 0.37 (0.16) [− 0.69; − 0.05]	−2.30	.022
Living together status ^d)^	−0.30 (0.16) [− 0.61; 0.02]	−1.86	.063	−0.24 (0.17) [− 0.57; 0.09]	−1.43	.153
Education ^e)^	−0.41 (0.16) [− 0.72; − 0.10]	−2.60	.009	−0.18 (0.12) [− 0.42; 0.05]	−1.55	.123

^a)^ 1 = male, 2 = female; ^b)^ 0 = not partnered, 1 = married/partnered; ^c)^ 0 = no children, 1 = children; ^d)^ 0 = living alone, 1 = living with somebody; ^e)^ 0 = secondary/advanced, 1 = university

## Discussion

### Major findings

This study investigated whether internal LoC and external LoC moderated the relationship between COVID-19 stress and general mental distress during the early months of the COVID-19 pandemic. Our major finding is that both aspects of LoC showed substantial moderation effects that proved to be robust in two different samples. In both the Norwegian and the German-speaking sample, LoC I served as a buffer of stress, while LoC E exacerbated stress. These results were found in unadjusted regression models and persisted in adjusted regression analyses. The association between COVID-19 stress and general mental distress was strong and pervasive, as indicated by significant conditional effects for the total range of scores of LoC I and LoC E. However, it was attenuated by the belief that the outcome of events in one’s life are contingent upon one’s actions (LoC I). On the contrary, the belief that chance and powerful others control one’s life (LoC E) even increased the already strong association between COVID-19 stress and mental distress. This suggests that the pandemic is particularly difficult to bear for those people who, in addition to any pandemic-related strains that may arise, feel that they have little influence on their own lives in general. The quasi-invisible and difficult to comprehend threat of a virus as well as restrictions on one’s own life determined by “powerful others” seem to reinforce a prevailing lack of self-control, as suggested by the positive correlation between COVID-19 stress and LoC E, and its negative correlation with LoC I. This was accompanied by drastically poorer mental health.

The opposite effect, which was found with a high internal locus of control, suggests that this is an important resource that should be focused on with regard to public health measures. The actions of political decision-makers can positively or negatively influence citizens’ experience of control, depending on the degree of involvement of representatives of different interest groups in decision-making processes [59]. Similarly, the form of policy communication is likely to have an impact on whether citizens perceive themselves as empowered or patronized, as has been evidenced in relation to young adults’ conflict strategies with superiors [60]. Last but not least, there are indications that a sense of control is associated with health behaviour and better health literacy [61, 62] - an aspect which, in the context of a pandemic, should not be separated from mental health.

Our results are consistent with the studies by Sigurvinsdottir et al. (2020) [40] and Alat et al. (2021) [41] who found moderate associations of higher external LoC and lower internal LoC, respectively, with mental distress during the COVID-19 pandemic. In the investigation by Berg & Lin (2020), COVID-19 preventative behaviors were not related to internal health-related LoC, but to external health-related LoC regarding powerful others [39]. It is beyond the scope of this article to discuss details of these counterintuitive results. However, it is important to keep in mind that the established mental health promoting effects of a high internal LoC and a low external LoC do not necessarily imply that these resources also promote preventive behavior that is based on self-restrictions and discipline. In terms of the current state of research, it can be said that, to our knowledge, until now the moderating role of LoC has not been investigated with respect to COVID-19 related outcomes. Our findings add to and confirm results of previous studies that established LoC as a factor that can maintain and improve (LoC I) or jeopardize (LoC E) mental health under stressful conditions [30, 34–38, 40, 41]. It can be assumed that locus of control and stress management interact in different ways. Early on, Rotter (1966) posited that the respective locus of control has an impact on how stress is perceived [29]. Individuals with an internal locus of control should be more likely to see difficult tasks as challenges rather than as something to be avoided. They should thus be more hopeful, active, and more likely to take responsibility for themselves and their environment. Correspondingly, LoC has been linked to different coping strategies [63]. A recent meta-analysis showed that internal LoC is indeed associated with adaptive coping (problem-focused and active strategies), and maladaptive LoC (excessive external locus and a lack of internal locus of control) with maladaptive coping, including avoidant and emotion-oriented strategies [37].

### The study samples in the context of COVID-19 mental distress research

Both study samples showed consistent major results, although they differed in their contextual background including pandemic stage, and demographic and psychological characteristics. On average, the Norwegian participants were older, better educated, more likely to be female and to have children. They also had better mental health, as indicated by less COVID-19 stress and lower rates of clinically significant general mental distress, also evidenced separately for depression and anxiety. An inspection of sample characteristics of recent psychological studies on pandemic-related moderator factors suggests that in the majority of these, participants were mostly young adults, more likely to be well-educated, and female [18–27]. Samples with high percentages of young adults and women also characterize epidemiological research on mental distress during the pandemic [2, 3, 6, 8] and the majority of studies of measurement of COVID-19 stress [11]. While both of our samples are comparable with these study characteristics concerning education and gender, the inclusion of middle-aged and older adults counterbalances the overrepresentation of younger adults in psychological COVID-19 research and offers a better generalisability over different age groups.

Concerning mental health, findings are available from recent systematic reviews and meta-analyses of mental distress in the general population at the beginning of the COVID-19 pandemic [1–8]. Compared with these synthesized prevalence data, the frequencies of clinically relevant depression, anxiety, and general mental distress in the German-speaking sample are in the middle range, and in the lower range in the Norwegian sample. Similar results were found in a study comparing mental health during the onset of the pandemic in Norway, Germany, and four other countries [43]. Prevalence of mental distress was higher in another Norwegian large-scale investigation that was carried out when all COVID-19 regulations were in force, and that was based on a sample with predominantly young adults [64]. Still, both the Norwegian and German-speaking samples presented here revealed degrees of mental distress that are higher than those reported in general population samples before the COVID-19 pandemic [49, 65–67].

### Elevated mental distress during the COVID-19 pandemic

There is ample evidence that mental disorders contribute to individual impairment and disability, as well as global burden of disease [68]. It is thus highly important to prevent COVID-19 stress and elevated mental distress from turning into pathology and mental disorders. On the other hand, elevated COVID-19 stress, and even temporarily increased symptoms of depression and anxiety can be regarded as functional psychological responses to a worldwide outbreak of a novel and life-threatening virus disease. Findings of strong stress reactions to a threatening situation should therefore not be “awfulized” by lurid headlines, which risks further exacerbating pathological developments (see [69]). We should also consider that data are still lacking on the long-term course of mental health after the COVID-19 pandemic. Preliminary follow-up data of the German-speaking sample suggest that mental distress increased directly after the first lockdown in spring 2020, and decreased slightly three months later, when the number of confirmed Sars-CoV-2 infections per million people in Europe was rather low [12, 70]. A recent large-scale study investigated differential trajectories of mental distress over eight weeks of full lockdown and subsequent eight weeks of easing of lockdown [71]. The authors found that previous mental health diagnoses, long-term health conditions, younger age, and lower incomes were among the strongest predictors of worse trajectories. While there are findings of significantly elevated mental distress even months and years after previous viral respiratory epidemics [72–74], these do not refer to the general population but to people who had personally experienced traumatic events, either as health care workers or as survivors of critical disease due to the respective respiratory syndromes.

### Limitations and strengths

The present study is based on two large samples from the general population which are, however, not representative. We accounted for this limitation by including important sociodemographic covariates in the analyses. Unadjusted as well as adjusted regression analyses yielded consistent results in both samples.

The 4-item Internal/External Locus of Control-4 Scale (IE-4) is a short scale, and questions may arise concerning its psychometric properties. In both samples of the present study, the two subscales showed McDonald’s omegas that were comparable with the omega values of the normative samples of the IE-4, suggesting sufficient reliability. For the German version, convincing data are reported regarding content, factorial, and construct validity [32]. The robust results of the current analyses also suggest sufficient validity of the IE-4.

It should be emphasised once again that our main outcome measure, the PHQ-4, does not establish diagnoses of mood or anxiety disorders according to ICD-10 or DSM-5. It measures core symptoms of both, thus indicating, by means of several cut-off scores, occurrence of clinically relevant symptoms. The PHQ-4 has been demonstrated to be a valid screening tool for general mental distress in the general population and clinical populations [e.g., 47–54, 66].

The COVID-19 stress scale was newly developed for the current investigation, as no instruments were available at the time we initiated the project [12]. In both samples of the present study, reliabilities were good, and results of confirmatory factor analyses suggest a one-dimensional model of COVID-19 stress. Its relationships with LoC, as well as with meaning in life, self-control, and crisis of meaning [12] corresponded to our hypotheses and can thus be considered as first evidence for construct validity.

## Conclusions

Our findings can offer important insights into how people with certain personality characteristics are well-equipped, whereas others are particularly vulnerable in times of crisis. According to the present study, people with an external locus of control are at special risk.

Decision-makers in the field of public health can take this into account. Improving citizens’ sense of control can help prevent increased mental distress from developing into mental disorders. Experiencing a sense of control may encourage citizens to adhere to necessary restrictions of individual freedom as a possible outcome of informed personal choice, rather than simply obeying an imposed rule. Possible ways of evoking a sense of control include clear, honest, and substantiated policy communication that is based on multiple perspectives, as well as explicit invitation of citizens to participate in decision-making, e.g. by expressing questions and objections. Although critical situations may require quick decisions and the short-term suspension of democratic processes, this should be done with utmost care, transparent explanation, and the quickest possible return to political action that seriously and credibly incorporates and implements citizens’ concerns. Feelings of stress caused by the pandemic on the one hand and of one’s own lack of control on the other hand obviously feed off each other, which is reflected in a worrying level of psychological distress.

## Supplementary Information

**Additional file 1:.** Supplementary Table S1: COVID-19 Stress scale.

## Data Availability

The datasets used and/or analysed during the current study are available from the corresponding author on reasonable request.
