# Supplementary material for: Locus of control moderates the association of COVID-19 stress and general mental distress: results of a Norwegian and a German-speaking cross-sectional survey
Source: BMC Psychiatry. 2021 Sep 6;21:437. doi: 10.1186/s12888-021-03418-5 (PMC8419811; doi:10.1186/s12888-021-03418-5)
Supplement: Supplementary file 1 — Additional file 1:. Supplementary Table S1: COVID-19 Stress scale. [file 12888_2021_3418_MOESM1_ESM.docx]

**Supplementary table S1: COVID-19 Stress scale**

**COVID-19 Stress scale** – German version*^+^

| Ich habe Angst vor dem, was durch die Pandemie auf uns zukommt. |
| --- |
| Ich glaube, ich werde die nächsten Monate gut durchstehen. (-) |
| Ich finde die derzeitige Situation unerträglich. |
| Ich fühle mich alleingelassen. |
| Ich langweile mich. |
| Ich bin ärgerlich. |
| Die Pandemie wird überall zu einer Verschlechterung der Lebensbedingungen führen. |

**Antwortformat:**

0 (stimme gar nicht zu) bis 5 (stimme vollständig zu)

**COVID-19 Stress scale** – Norwegian version^+^

| Jeg er redd for pandemien og hva som kan skje med oss. |
| --- |
| Jeg tror jeg skal komme meg godt gjennom de neste månedene. (-) |
| Jeg synes dagens situasjon er uutholdelig. |
| Jeg føler meg overlatt til meg selv. |
| Jeg kjeder meg. |
| Jeg er irritert. |
| Jeg tror koronakrisen vil føre til en forverring av levekårene for alle. |

**Svarformat:**

0 (stemmer ikke) til 5 (stemmer helt)

**COVID-19 Stress scale** – English version^#^

| I am afraid of the pandemic and what it will bring. |
| --- |
| I think I will get through the next few months in good shape. (-) |
| I find the current situation unbearable. |
| I feel left alone. |
| I am bored. |
| I am annoyed. |
| The pandemic will lead to a deterioration of living conditions all over. |

**Response format:**

0 (strongly disagree) to 5 (strongly agree)

^+^ Krampe, H., Danbolt, L. J., Haver, A., Stålsett, G., & Schnell, T. (under review). Locus of control moderates the association of COVID-19 stress and general mental distress: Results of a Norwegian and a German-speaking cross-sectional survey.

* Schnell, T. & Krampe, H. (2020). [Meaning in life and self-control buffer stress in times of COVID-19: Moderating and mediating effects with regard to mental distress.](https://www.frontiersin.org/articles/10.3389/fpsyt.2020.582352/full?&utm_source=Email_to_authors_&utm_medium=Email&utm_content=T1_11.5e1_author&utm_campaign=Email_publication&field=&journalName=Frontiers_in_Psychiatry&id=582352) *Frontiers in Psychiatry, 11*:582352. doi: 10.3389/fpsyt.2020.582352

^#^ Translation
